# Feasibility of the evidence-based cognitive telerehabilitation program *Remind* for patients with primary brain tumors

**DOI:** 10.1007/s11060-017-2738-8

**Published:** 2018-01-10

**Authors:** Sophie D. van der Linden, Margriet M. Sitskoorn, Geert-Jan M. Rutten, Karin Gehring

**Affiliations:** 1 Department of Neurosurgery, Elisabeth-TweeSteden Hospital, Tilburg, The Netherlands; 2 Department of Cognitive Neuropsychology, Tilburg University, P.O. Box 90153, 5000 LE Tilburg, The Netherlands

**Keywords:** Cognitive rehabilitation, Telerehabilitation, eHealth, Glioma, Meningioma, Neurosurgery

## Abstract

Many patients with primary brain tumors experience cognitive deficits. Cognitive rehabilitation programs focus on alleviating these deficits, but availability of such programs is limited. Our large randomized controlled trial (RCT) demonstrated positive effects of the cognitive rehabilitation program developed by our group. We converted the program into the iPad-based cognitive rehabilitation program *ReMind*, to increase its accessibility. The app incorporates psychoeducation, strategy training and retraining. This pilot study in patients with primary brain tumors evaluates the feasibility of the use of the *ReMind*-app in a clinical (research) setting in terms of accrual, attrition, adherence and patient satisfaction. The intervention commenced 3 months after resective surgery and patients were advised to spend 3 h per week on the program for 10 weeks. Of 28 eligible patients, 15 patients with presumed low-grade glioma or meningioma provided informed consent. Most important reason for decline was that patients (7) experienced no cognitive complaints. Participants completed on average 71% of the strategy training and 76% of the retraining. Some patients evaluated the retraining as too easy. Overall, 85% of the patients evaluated the intervention as “good” or “excellent”. All patients indicated that they would recommend the program to other patients with brain tumors. The *ReMind-*app is the first evidence-based cognitive telerehabilitation program for adult patients with brain tumors and this pilot study suggests that postoperative cognitive rehabilitation via this app is feasible. Based on patients’ feedback, we have expanded the retraining with more difficult exercises. We will evaluate the efficacy of *ReMind* in an RCT.

## Introduction

Many patients with primary brain tumors suffer from cognitive deficits [1, 2]. These deficits can cause difficulties in patients’ everyday lives and affect their quality of life [3, 4]. Reported prevalence rates of cognitive deficits vary widely, which is partly due to the differences in used methods, but range between 19 and 90% [2, 5, 6]. Since survival rates are increasing [7, 8] and patients are living longer with possible cognitive deficits, management of cognitive deficits becomes an increasingly important part of total care in patients with primary brain tumors.

Unfortunately, treatment options for these cognitive deficits are scarce. Over the last years, a few intervention studies have been conducted in brain tumor patients, which demonstrated positive effects of cognitive rehabilitation [9–11]. Our randomized controlled trial (RCT) in 140 glioma patients with stable disease demonstrated positive effects of a 6-week face-to-face cognitive rehabilitation program that consisted of psychoeducation, teaching of use of compensatory skills and retraining [9, 12]. Despite the positive findings of previous studies and patients’ needs for rehabilitation services, cognitive rehabilitation is not always accessible for every patient in clinical practice [13, 14]. Conventional in-person cognitive rehabilitation can be demanding and costly, due to, amongst others, multiple hospital visits and lengthy face-to-face sessions with professionals.

To overcome some of the limitations of conventional cognitive rehabilitation, a number of studies explored the possibilities of cognitive telerehabilitation programs in other neurological and oncological patient populations [15–18]. Cognitive telerehabilitation is a form of eHealth, and it is defined as “the use of information and communication technologies to provide rehabilitation services to people remotely in their homes or other environments” [19]. To our knowledge, no studies on cognitive telerehabilitation have been conducted in adult patients with brain tumors.

Based on the positive findings of our previous RCT, and ongoing requests of doctors and patients to utilize the cognitive rehabilitation program, we converted our program into an iPad-based cognitive rehabilitation application, named *ReMind*. The goal of the development of the *ReMind*-app was to increase the accessibility of the program to brain tumor patients in a cost-efficient mode of delivery, while maintaining the contents of the original program. Before we initiated an RCT to evaluate the efficacy of *ReMind*, we conducted a small-scale study to investigate the feasibility of, and potential barriers to, the use of the program in the clinical (research) setting in terms of accrual, attrition, adherence and patient satisfaction. Since cognitive telerehabilitation has not yet been investigated in adult patients with primary brain tumors (i.e. vulnerable patients with higher levels of fatigue, psychological distress and concentration problems), this feasibility study is an important first step.

## Methods

### Participants

Patients with a radiologically suspected supratentorial low-grade glioma or meningioma, who were scheduled for resective surgery in the Elisabeth-TweeSteden Hospital Tilburg, were invited to participate. Patients who met any of the following criteria were excluded: history of intracranial neurosurgery, history of severe psychiatric or neurological disorder, diagnosis of multiple meningioma, complete unfamiliarity with the use of computers, lack of basic proficiency in Dutch, inability to undergo neuropsychological assessment due to motor/language/visual problems, Karnofsky performance score (KPS) below 70 or a premorbid IQ below 85. Patients who were referred to in- or outpatient cognitive rehabilitation were excluded as well. The projected sample size was 15. Informed consent was obtained from all individual participants included in the study.

### Design and procedure

This single-arm pilot study was approved by the local medical ethical review board (METC Brabant: NL51152.028.14), registered in The Netherlands National Trial Register (NTR 5392).

Two weeks before surgery, patients were informed about the study by a nurse practitioner. Interested patients received an information letter. One day before surgery, patients were hospitalized and neuropsychological assessment (T0) was carried out as part of usual clinical care. At the beginning of the assessment, patients who were willing to participate in the study provided written informed consent. If patients chose to make use of the possibility to involve a significant other in the cognitive rehabilitation trajectory (see below), the significant other had to give informed consent as well. Three months after surgery, a second usual care neuropsychological assessment (T3) was conducted. Immediately afterwards, the cognitive telerehabilitation program *ReMind* commenced. Three months later (i.e. 6 months after surgery), after completing *ReMind*, the final neuropsychological assessment took place (T6) for the purpose of this study. Additionally, study-specific evaluation questionnaires were completed. The current study was embedded in standard clinical care provided by the hospital.

### Intervention

#### The program

**Fig. 1:**
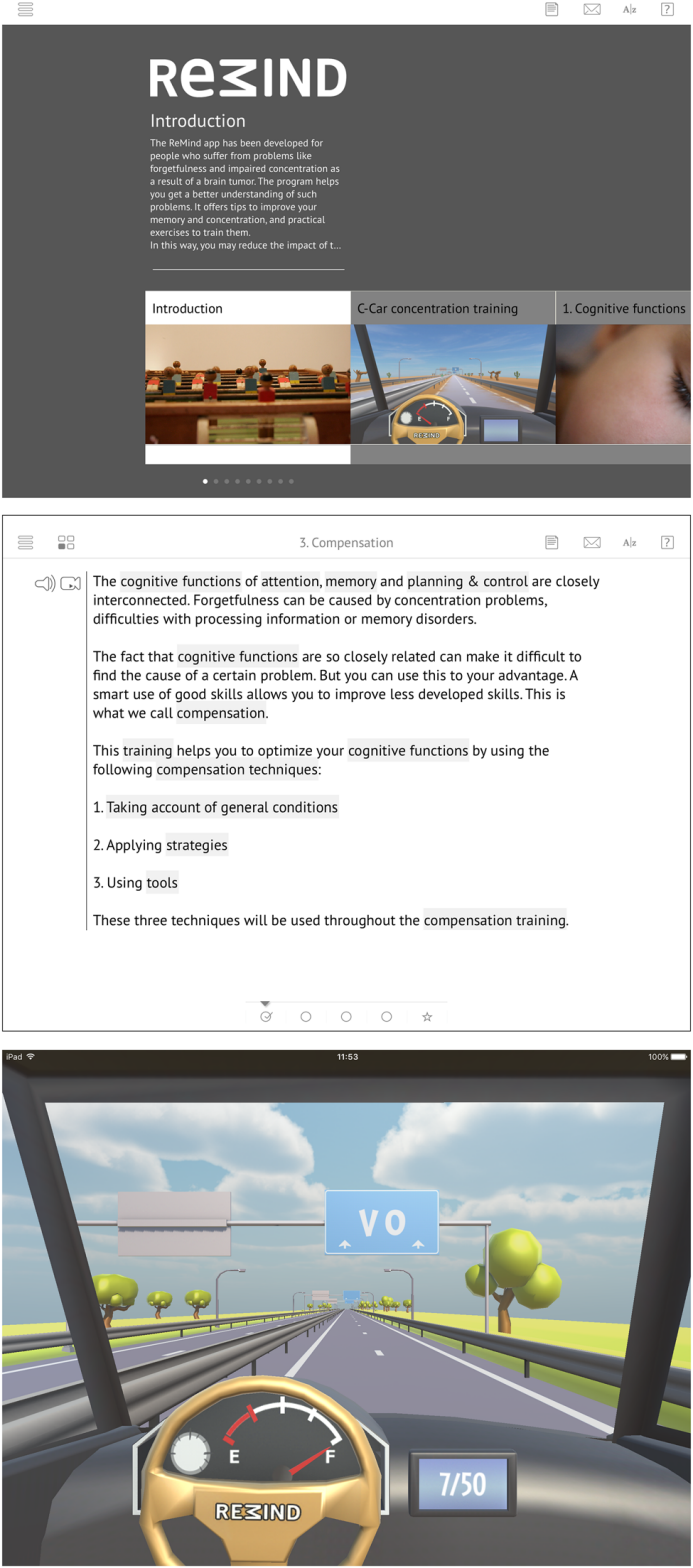
Screenshots of different parts of *ReMind*: **a** homepage, **b** strategy training, and **c** retraining

The cognitive telerehabilitation program *Remind* was developed in a joint patient/researcher initiative and is based on our previously evaluated face-to-face cognitive rehabilitation program [9, 12, 20]. The *ReMind*-app is provided via an iPad (Fig. 1a) and is available in both Dutch and English. In the current study, the Dutch version was used.

Similar to the original program [12], *ReMind* consists of compensation training, including psychoeducation and teaching of compensatory skills, and attention retraining (see Fig. 1b, c). In the compensation training, psychoeducation about cognitive functions is provided in six modules, namely on (1) Cognitive functions, (2) Influences, (3) Compensation, (4) Attention, (5) Planning & control, and (6) Memory. Additionally, in each module, compensatory strategies are taught and many exercises are included to learn to apply these strategies in everyday life. Patients learn, for example, to minimize distraction and deal with time pressure, and to optimally use external devices for support. Due to the strong interdependence of all cognitive functions, the strategy training was designed so that patients should go through all the six modules one by one, to benefit the most from the strategy training. Progression through each module is visualized, with checkmarks at the bottom of the screen (Fig. 1b).

In the retraining part, named *C-Car*, four different modes of attention are trained, namely sustained, selective, alternating and divided attention. It includes visual and auditory exercises, wherein both verbal and numeric stimuli are presented. All patients started with the same version of this training, independently of their pre-intervention neuropsychological scores. Series of hierarchically graded tasks were used, so that higher levels are reached, if previous levels are mastered. In this manner, the retraining is tailored to the level of the patient. After each exercise, patients receive feedback on their performance.

During the development of the app, optimum use was made of the additional (technical) possibilities the new environment offered. The instructional texts of the strategy training are provided in videos, audio clips and read-only formats and patients can look back as often as they feel necessary. *ReMind* incorporates several other functions to make it as user-friendly as possible, such as help-overlay screens and links to explanations of important definitions. The program also offers the possibility to involve a significant other, which can be a spouse, family member, friend or professional: the *ReMinder*. Patients can send this *ReMinder* an email from anywhere in the program, for example to ask for advice when they get stuck in a text or an exercise.

#### Guidance

Three months after surgery, immediately after the second neuropsychological assessment (T3), a face-to-face appointment was planned, to hand over the iPad on which the *ReMind*-app was installed together with an explanation of the app. During the intervention period, the researcher contacted the patients by telephone every 2 weeks, to check on their progress, plan the course of their training and to address questions. It was advised and expected that patients spent 3 h per week on the program, to complete the program within 10 weeks. A second face-to-face appointment took place at the end of the program, to retrieve the iPad and to collect the completed questionnaires.

### Measures

#### Accrual and attrition

Accrual was defined as the total number of included patients as compared to the number of invited patients. The number of patients who declined participation and reasons for decline were carefully recorded. The same was done for the number of patients who dropped out of the study and reason(s) for this attrition.

#### Adherence

Adherence to the program was indicated by both the number of completed module sections in the strategy training and the number of exercises performed in the retraining, each expressed in percentages of total available sections and exercises, respectively. If patients completed ≥ 80% of both the strategy training and the retraining, adherence was considered acceptable. To calculate mean percentages for the group, a maximum of 100% per individual was used, even if patients worked through the program more than once. Reasons for non-adherence as reported in the telephone calls during the intervention and in the face-to-face appointment at the end of the program were recorded.

#### Patient experience

After completing the program, patients were requested to fill out a study-specific questionnaire, evaluating their experiences with *ReMind* (e.g., satisfaction, enjoyment, usefulness and burden), whether they would recommend any changes in (elements of) the program, and if they would recommend it to other patients.

#### Feasibility of neuropsychological assessments

Neuropsychological tests and patient-reported outcome measures (PROMs) were administered to describe baseline functioning of the patients and to test the feasibility of procedures for later use on a larger scale. Objective cognitive functioning was assessed by the computerized neuropsychological test battery CNS Vital Signs [21, 22] and three paper-and-pencil tests, namely Letter Fluency, Digit Span (WAIS), and Paired Associates (WMS) [23–25]. *Z*-scores were calculated using normative data and *Z*-scores ≤ − 1.5 were considered as low. Subjective cognitive functioning was assessed with the Cognitive Failures Questionnaire (CFQ) [26]. Based on Dutch representative normative data [27], a total score of ≥ 42 was considered as clinically high. Symptoms of anxiety and depression were assessed with the Hospital Anxiety and Depression Scale (HADS) [28, 29], with a cut-off for both scales of ≥ 8.

### Data analysis

Percentages of eligible, included, excluded and dropped-out patients were calculated. Descriptive statistics of participants are presented. This feasibility study (*n* = 15) was not designed, and therefore not powered, to evaluate the efficacy of *ReMind*.

## Results

### Accrual and attrition

**Fig. 2:**
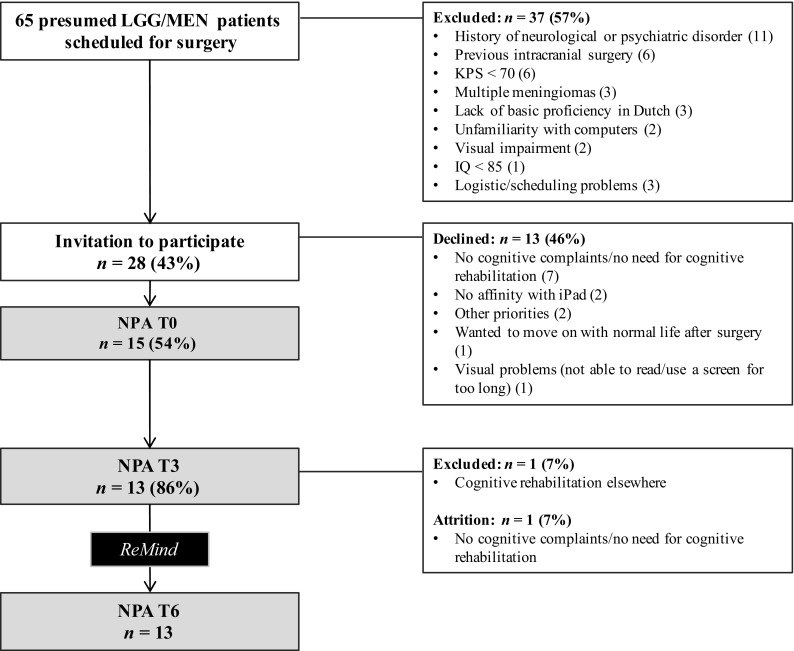
Flowchart of enrolment and attrition. *NPA* neuropsychological assessment, *LGG* low-grade glioma, *MEN* meningioma, *KPS* Karnofsky performance score

Data on accrual and attrition are presented in a flow diagram (Fig. 2). Out of 65 consecutive patients who were scheduled to undergo surgery for presumed low-grade glioma/meningioma, 37 patients (57%) were excluded. Of the 28 eligible patients who were invited to participate, 15 patients provided informed consent (54%) and 13 patients (46%) declined. The most important reason for decline was that patients (*n* = 7) did not experience cognitive deficits and felt no need to follow a cognitive rehabilitation program at this stage.

In the 3 months prior to the start of the intervention, one patient withdrew from the study because of lack of cognitive complaints. An additional patient was excluded after informed consent, since she was referred to cognitive rehabilitation elsewhere. No dropout occurred during the intervention phase. Nine participants (69%) chose to involve a significant other (in all cases, a spouse).

### Demographic, clinical and neuropsychological characteristics

Table 1 shows the demographic, clinical and neuropsychological characteristics of the sample. Thirteen patients (38% female), with a mean age of 52 years (range 40–68), followed the cognitive rehabilitation program and completed all assessments. Six patients were diagnosed with a grade I meningioma, one patient with a WHO grade II meningioma and four patients with a WHO grade II glioma. For two other patients, the radiologically suspected diagnosis of low-grade glioma was not confirmed after surgery (Table 1). After surgery, five patients (38%) were treated with radiotherapy and three of these patients also received chemotherapy at T3. The majority (69%) of the patients were highly educated. Before the start of the intervention, seven patients demonstrated low *Z*-scores (≤ − 1.5) on one or more measures of objective cognitive functioning. Based on the scores of the HADS, three of the patients possibly suffered from depression, and one of them possibly from anxiety as well (Table 1).

**Table 1:** Characteristics and adherence per participant

	Sex	Age	Education (years; level^a^)	Histology	Tumor hemisphere	Tumor location	RTx	CTx	AED	OCF^b^ at T3	PROM^c^ at T3	Significant other involved	Strategy training completed (%)^d^	Retraining completed (%)^d^	Sufficient adherence^d^	Reported difficulties^e^
1	♂	68	17; 6	MEN	Left	Frontal	No	No	No	2	No	Yes	83	100	Yes	A
2	♀	54	15; 6	MEN	Bilateral	Frontal	No	No	No	0	No	No	62	178	No	A
3	♂	53	18; 6	MEN	Right	Temporal	No	No	Yes	0	No	No	100	56	No	B/C
4	♀	43	15; 6	MEN	Right	Frontal	No	No	No	1	No	No	17	15	No	D
5	♂	54	15; 5	Inconcl	Right	Parieto-occipital	No	No	Yes	4	An + D	Yes	100	81	Yes	
6	♂	59	13; 5	GBM	Right	Insular	Yes	TMZ	Yes	0	No	Yes	42	100	No	
7	♂	44	16; 6	LGG^f^	Left	Temporal + insular	Yes	No^g^	No	1	No	Yes	100	131	Yes	
8	♂	61	16; 6	LGG^f^	Right	Frontal	Yes	TMZ	Yes	5	D	Yes	83	84	Yes	E/F
9	♀	43	13; 4	MEN	Bilateral	Frontal	No	No	Yes	3	No	No	17	13	No	E
10	♂	56	17; 6	MEN-II	Right	Frontal	Yes	No	Yes	0	No	Yes	100	100	Yes	E
11	♂	40	20; 6	LGG^f^	Left	Temporal	Yes	TMZ	Yes	0	No	Yes	100	103	Yes	
12	♀	52	14; 5	MEN	Bilateral	Frontal	No	No	No	0	No	Yes	17	31	No	D
13	♀	47	18; 6	LGG	Right	Frontal	No	No	Yes	1	D	Yes	100	100	Yes	
Total	♂:8♀:5	Mean 52	Median 16; 6	MEN: 7 LGG:4	L: 3|R: 7 Bilateral: 3	Frontal: 8 62%	5 38%	3 23%	8 62%	7 54%	3 23%	9 69%	Mean (median) 71 (83)	Mean (median) 76 (100)	7 54%	

*RTx* radiotherapy prior or during *ReMind, CTx* chemotherapy prior or during *ReMind, AED* anti-epileptic drugs, *OCF* objective cognitive functioning, *PROM* patient-reported outcome measure, *MEN* meningioma (WHO-grade I), *MEN-II* WHO-grade II meningioma, *LGG* low-grade glioma, *GBM* glioblastoma multiforme, *TMZ* temozolomide, *An* anxiety, *D* depression

^a^Education is classified according to the Dutch coding system of Verhage ranging from 1 (less than primary education) to 7 (university degree) (Verhage, 1964)

^b^Number of impaired outcomes of objective cognitive functioning (*Z*-score of ≤ − 1.5; 11 scores were considered)

^c^PROMs included the CFQ and HADS, with cut-offs of ≥ 42 and ≥ 8 respectively, ‘No’ indicated no scores above the cut-offs

^d^To calculate mean percentages, a maximum of 100% per individual was used. Completion of ≥ 80% of both the strategy training and retraining was considered as sufficient

^e^*A* illness of spouse, *B* technical problems, *C* return to full-time work, *D* other priorities, *E* rehousing, *F* severe fatigue due to adjuvant treatment

^f^Awake craniotomy

^g^Received temozolomide after T6

### Adherence

Adherence per participant is presented in Table 1. On average, participants completed 71% of the strategy training 76% of the retraining. According to our definition of adherence (completion of ≥ 80% of both the strategy training and the retraining), seven out of 13 patients (54%) adhered to the program. Six patients (46%) completed the entire strategy training and seven patients (54%) completed the entire retraining. Four patients (31%) fully completed both the strategy- and retraining. Three participants reported specific circumstances that explained non-adherence: one was confronted with serious illness of her spouse, one experienced technical problems with the retraining part of the program (which were solved afterwards) and one moved to a new house during the intervention. Two participants who had a (very) low adherence to the program reported that they were too busy with other activities and had other priorities. On the other hand, other patients also experienced interfering circumstances, but were still able to adhere to the intervention. One low-grade glioma patient reported that following the program was burdensome in combination with adjuvant tumor treatment.

### Patient experience

Results of the study-specific evaluation questionnaire are listed in Table 2. The majority evaluated the difficulty and the quantity of psychoeducation, fill-in exercises (to practice with learned strategies) and retraining tasks as sufficient. However, four participants reported that there were too many fill-in exercises included in the strategy training, whereas four other participants rated the retraining as (a bit too) easy and found there were (too) few retraining exercises included in the program. Furthermore, eight patients enjoyed working with *ReMind*. Using an iPad-app for cognitive rehabilitation was appreciated. Overall, 11 patients (85%) evaluated the cognitive rehabilitation program *ReMind* as “good” or “excellent”. All participants indicated that they would recommend the program to other brain tumor patients.

**Table 2:** Post-intervention ratings of different aspects of *ReMind* (*n* = 13)

Difficulty of	(Too) easy	Just right	(Too) difficult
Information in strategy training	3	10	–
Fill-in exercises in strategy training	1	10	2
Retraining (*C-Car* game)	4	9	–
Amount/number of	(Too) little/few	About right	(Too) much/many
Information in strategy training	1	12	–
Fill-in exercises in strategy training	–	9	4
Retraining exercises (*C-Car* game)	4	8	1
Supervision of the researcher/trainer	–	13	–
Usefulness of	(Very) useful	Neutral	Not useful
Information in strategy training	7	6	–
Fill-in exercises in strategy training	3	9	1
Retraining exercises (*C-Car* game)	11	2	–
(Telephone) contact with the researcher/trainer	13	–	–
Content addressed daily problems	Fully/largely	Partly	Not
	8	4	1
Application of learnt (strategies) in daily life	Often/regularly	Sometimes	Seldom/never
	3	5	5
Impact of cognitive problems has changed	Yes, positively	No^a^	Yes, negatively
	6	7	–
Coping with cognitive problems has changed	Improved coping	No^b^	Worsened coping
	5	8	–
Pleasantness of working on *ReMind*	(Very) pleasant	Neutral	(Very) unpleasant
	8	3	2
	Excellent/good	Sufficient	Insufficient/poor
Using an iPad-app for cognitive rehabilitation	11	1	1
Capability of the researcher/trainer	13	–	–
Contact with the researcher/trainer	12	1	–
Overall rating of the program	11	1	1
	Yes	No	
Recommendation to other brain tumor patients	13	0	

^a^No change, there was no impact on daily life (5) or no change, impact remained the same (2)

^b^Coping is still good (8), or coping is still not good (0)

### Feasibility of neuropsychological assessments

Three patients were excluded from the study beforehand, because they did not undergo the first neuropsychological assessment (T0) due to (logistical) problems with planning. All 13 participants fully completed neuropsychological assessments, one questionnaire of a participant was not returned.

## Discussion

*ReMind* is the first cognitive telerehabilitation program specifically developed for adult patients with primary brain tumors. The current pilot study was designed to test the feasibility of an evidence-based telerehabilitation program in the clinical (research) setting in preparation for a larger RCT. The results suggest that, for the subset of interested patients who were included in the study based on specific criteria, cognitive rehabilitation by using the *ReMind*-app was feasible. Overall, participants were satisfied with the program and dropout was low.

The recruitment of participants to the study was the most challenging aspect of this feasibility study. A substantial part (57%) of the patients who were undergoing surgery for low-grade glioma or meningioma were not eligible based on the exclusion criteria. In hindsight, the exclusion criteria appeared to be overly strict, which, in an attempt to reduce bias through controlling patients’ characteristics and potential confounders, potentially compromised the generalizability of the results to the target patient population of patients with presumed low-grade glioma and meningioma [30, 31]. In particular, a large proportion of patients with a history of neurological/psychiatric disorders or previous intracranial surgery were excluded, although cognitive rehabilitation may be relevant for them as well. Based on these experiences, we adapted the inclusion criteria of the RCT. Furthermore, 46% of eligible patients declined participation, half of them reporting to feel no need to undergo cognitive rehabilitation at this stage (*n* = 7). Two patients specifically declined participation since an iPad-based intervention was not appealing to them.

With respect to adherence, 54% of the participants met the criterion for sufficient (completion of ≥ 80% of both the strategy training and the retraining) adherence, which was comparable with other studies that investigated psychological eHealth interventions in other patient populations [32], but not as high as the adherence to the face-to-face program in our previous RCT [9, 12]. Whereas our previous study included a sample of patients with clinically stable lower-grade gliomas with a disease duration of several years, the patients in the present study participated only shortly after, or even during, the tumor treatment phase of their disease. For example, five patients received adjuvant treatment during the intervention. The patients in our pilot study lived through a turbulent period, in which psychosocial developments, in addition to medical treatment and recovery, are predominating. Some of them resume their work and/or family care during this period. It may be that, for some patients, undergoing cognitive rehabilitation in this phase is (too) burdensome. Furthermore, in contrast to our previous study, experiencing subjective cognitive (or objective) dysfunction was not an inclusion criterion. Consequently, not all patients in our sample experienced cognitive dysfunction (yet), which may have led to a lack of motivation to fully adhere to the program for some. Along this line, all three participants who reported psychological complaints showed (more than) sufficient adherence rates.

In order to reduce the well-known problems with adherence in remote interventions and, in particular, to find substitutes for the low amount of (face-to-face) supervision, before the start of the pilot study, we incorporated several features into the program that are known to enhance adherence [32–34]. For example, we provided regular guidance during the intervention, through telephone counseling and by provision of feedback from the program itself. Additionally, the program offers the possibility to patients to involve a significant other in the process, an option that nine patients chose. Seven of these patients showed sufficient adherence rates. Despite the efforts made, adherence rates were suboptimal.

Overall, participants who followed the program reported that an iPad-app was an appropriate mode of delivery of cognitive rehabilitation. In fact, this mode enables many patients with brain tumors to follow a cognitive rehabilitation program at their own homes, which is a great advantage since many patients are not allowed to drive due to epileptic seizures. Another important advantage is that patients can follow the program at their own pace and can spread the material over as many sessions as they want, which could be helpful for (vulnerable and/or) older patients in particular [20]. All patients indicated that they would recommend to program to other brain tumor patients. However, some participants indicated that the retraining was too easy for them. Therefore, we decided to expand the retraining with nine more difficult exercises for use in the RCT and beyond, ensuring that the retraining remains challenging for each individual.

In intervention studies, the timing of the intervention is an important, but difficult issue, wherein a balance is sought between intervening not too early, but also not too late. Research in patients with brain tumors demonstrated that the need for supportive care is very high, especially in the early stage of the disease [14]. We hypothesized that early cognitive rehabilitation may enhance the recovery process and may prevent/minimize the negative impact of cognitive side effects of adjuvant treatment, and we decided to start the intervention soon after physical recovery from the surgery and after completion of radiotherapy. At three months after surgery, the intervention could be easily embedded in the existing logistics of our clinical aftercare, thereby minimizing patient burden. Because of the hypothesized preventative effects at this stage, both patients with and without cognitive complaints/deficits were eligible. We assume that several aspects of our cognitive rehabilitation program, for example psychoeducation about cognitive (dys)functioning in patients with brain tumors, could be helpful for a broad group of patients at an early stage.

We have started a larger trial to evaluate the efficacy of *ReMind* with respect to cognitive functioning and patient-reported outcomes, in which patients are consecutively randomized to an intervention group or waiting-list control group by minimization [35] after the 3 months’ assessment. Based on the experiences in the pilot study, exclusion criteria were revised to include a broader group of patients and two participating medical centers were added. A 6-month follow-up assessment was added to the design and participants are requested to keep records of their time spent on the program using registration forms. In addition, a pilot study in 20 glioma patients with stable disease and cognitive complaints is currently being conducted, using the English version of the *ReMind*-app, at the University of California, San Francisco (UCSF) (clinicaltrials.gov NCT02783495). Ultimately, if the results of the RCT demonstrate beneficial effects of *ReMind* at the postoperative stage, this telerehabilitation program may enable many patients with brain tumors to follow a cognitive rehabilitation program at their own pace in their own environment early in the course of the disease.
